# Mediation effect of plasma metabolites on the relationship between immune cells and the risk of prostatitis: A study by bidirectional 2-sample and Bayesian-weighted Mendelian randomization

**DOI:** 10.1097/MD.0000000000040024

**Published:** 2024-10-11

**Authors:** Chao Ding, Quanhua Gong, Shui Wan

**Affiliations:** a Department of Urology, Wuhu Hospital of Traditional Chinese Medicine, Wuhu, Anhui Province, China; b Department of Urology, The Affiliated Hospital of Anhui College of Traditional Chinese Medicine, Wuhu, Anhui Province, China.

**Keywords:** Bayesian-weighted Mendelian randomization, immunophenotypes, mediation, metabolites, prostatitis

## Abstract

According to the findings of multiple observational studies, immune disorder was a risk factor for prostatitis. However, it remained unknown whether there was a direct causal relationship between immune cells and prostatitis or whether this relationship was mediated by plasma metabolites. Based on the pooled data of a genome-wide association study (GWAS), a genetic variant was used to predict the effects of 731 immunophenotypes on the risk of prostatitis and determine whether the effects were mediated by 1400 metabolites. The bidirectional 2-sample Mendelian randomization (MR) method was adopted to uncover the causal relationship between immunophenotypes and prostatitis. Subsequently, a 2-step MR method was employed to evaluate whether the metabolites mediated this causal relationship and quantify the mediating effects and the corresponding ratios. In addition, the Bayesian-weighted Mendelian randomization (BWMR) method was employed to verify the results. Among the 731 immunophenotypes analyzed, 16 had causal relationships with the risk of prostatitis, including 11 with positive correlations (*P* < .05, beta > 0) and 5 with negative correlations (*P* < .05, beta < 0). The MR analysis screened out 9 metabolites related to the risk of prostatitis. The X − 24344 levels mediated the causal relationship between CD3 on CD39^+^ activated Treg and prostatitis (mediation effect: 0.01; ratio: 9.82%). Both histidine betaine (hercynine) levels and the proline-to-glutamate ratio mediated the causal relationship between CD14^–^CD16^+^ monocyte absolute count and prostatitis, with the mediation effects of −0.016 (14.20%) and −0.008 (7.24%), respectively. The glutamine degradant levels mediated the causal relationship between HLA DR^+^ CD4^+^ %T cells and prostatitis, with a mediation effect of −0.012, accounting for 8.07% of the total. The present study indicated that the immune cell subsets predicted based on gene expression profiles were potentially beneficial or harmful risk factors of prostatitis, and plasma metabolites may serve as the mediating factors of the relationship. The study thus shed light on deciphering the immunologic mechanism of prostatitis.

## 1. Introduction

Prostatitis, the most common disease in males, is characterized by complicated clinical symptoms including pain of the perineum, paruria, sexual dysfunction, male infertility, and psychological illness, seriously affecting the patients’ physical and psychological health. Researchers have identified 4 types of prostatitis: Type 3 was chronic prostatitis/chronic pelvic pain syndrome (CP/CPPS), which belonged to aseptic inflammation and was the most common type clinically, with an incidence of 90% to 95%; types 1 and 2 were bacterial prostatitis with a low incidence (5–8%), primarily caused by pathogen infections; type 4, asymptomatic inflammatory prostatitis, did not exhibit any clinical symptoms and was diagnosed only occasionally.^[1,2]^

CP/CPPS, a complex syndrome with unknown causes and pathogenesis, might be associated with immunity, trauma, psychological factors, and nervous and endocrine system dysfunction and was difficult to be cured.^[1]^ Increasing evidence demonstrated that it was an autoimmune disease, with the occurrence associated with immune disorder.^[3–5]^ The proportions of effector T cells including Th1, Th17, and Th22 cells in the peripheral blood of such patients were significantly higher than those in the population without CP/CPPS,^[6]^ and the levels of IgG and CD8^+^ T cells showed significant differences between the diseased and healthy populations.^[7]^ Macrophage migration in the prostate aggravated the inflammatory infiltration and pain in the patients,^[8]^ while Th17 cell-mediated immune response increased the risk of CP/CPPS.^[9]^ Nevertheless, these studies had insufficient samples and were easily affected by confounding factors that might cause biases, distortion, and reverse causal relationships. In addition, because of diverse factors including ethical requirements for the experiments and differences in subjects, types, and methodology, the definite immunopathogenesis and key targets of CP/CPPS were not identified in these studies. Therefore, effective treatment suggestions fail to be provided based on high-level evidence.

Metabolic remodeling is a key feature of immune cell proliferation, activation and differentiation.^[10]^ To gain a deeper understanding of the features of immune system disorder in patients with prostatitis, we further investigated the metabolic characteristics of the immune response. According to existing evidence, acetylation of acetyl coenzyme A derived from glucose, fatty acids and acetate was conducive to maintaining the survival, functioning and differentiation of T cells,^[11]^ suppressed the production of Th17 cells with inflammatory response by inducing the glycolysis-fat metabolism process of effector T-cell population, and promoted the production of regulatory T cells with anti-inflammatory action (Tregs).^[12,13]^ When glutamine metabolism process of naive CD4^+^ T cell population was deprived, naive T cells were found to have an accelerated differentiation to Tregs.^[14]^ Some metabolic pathways also affected the immune response of macrophages to change the homeostasis of immune microenvironment. Glucose uptake and glycolysis accelerated the transformation of macrophages to pro-inflammatory M1 macrophages, for example, all the metabolites involved in tricarboxylic acid cycle (citric acid, isocitric acid, succinic acid, fumaric acid, and malic acid) induced the production of M1 macrophages.^[15]^ Some amino acid metabolism pathways, fat oxidation and oxidative phosphorylation pathway contributed to the production of anti-inflammatory M2 macrophages, for example, the polarization of M2 macrophages can be upregulated by high levels of *α*-ketoglutarate/succinic acid, histone acetylation involving citrate and acetyl coenzyme A, and polyunsaturated fatty acid.^[16]^ In addition, dendritic cells (DCs) play crucial roles in both the immunogenicity and immune tolerance in different metabolic reprogramming processes. Both glycolysis and fatty acid synthesis enhance the pro-inflammatory response and migratory capacity of DCs. In contrast, oxidative phosphorylation and the metabolism of Vitamin D3 and certain amino acids (L-tryptophan and L-arginine), which are actively degraded by idoleamine-2,3 dioxygenase (IDO1) and arginase, result in the production of tolerogenic DCs.^[17]^ These findings underscore the significant role of metabolites in regulating immune homeostasis. However, it remains unclear whether these metabolic processes contribute to the immune imbalance observed in patients with prostatitis.

Mendelian randomization (MR) is a time-saving and cost-effective statistical method for epidemiology. It adopts pooled large-scale GWAS data to screen the instrumental variables (IVs) and deduce the causal relationship between the pathogenesis (or risk factors) and the disease.^[18]^ The genetic variant (allele) was assigned randomly in the interkinesis process and not susceptible to diseases. Therefore, the study can be called a natural randomization control study.^[19]^ This study adopted the MR method to explore the causal relationship between 731 immunophenotypes and prostatitis in the human body. Furthermore, this study probed into the complicated pathogenesis of prostatitis and discussed whether the relationship was mediated by 1400 metabolites. Meanwhile, we mined potential therapeutic targets and gained clues in the prevention and treatment of prostatitis by intervening in immune cells and mediating metabolites.

## 2. Materials and methods

### 2.1. Study design

**Figure 1.**
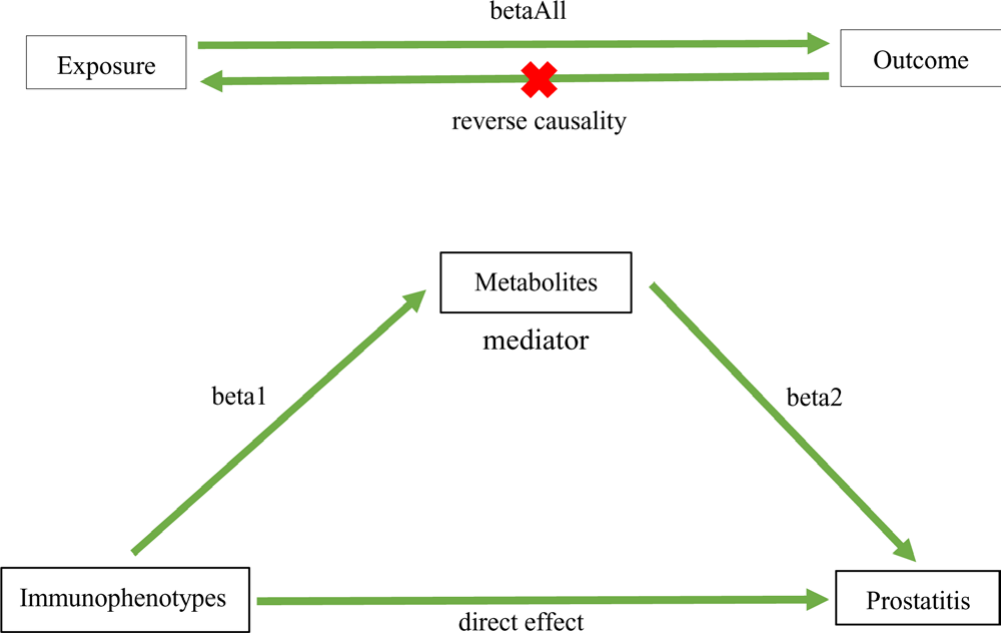
A draft of the causal relationship between immunophenotypes and prostatitis mediated by metabolites. Immunophenotypes as exposure, prostatitis as outcome, and metabolites as mediator. beta1 is the causal effect of exposure to mediator, beta2 is the causal effect of mediator to outcome, and betaAll is the total causal effect of exposure to outcome.

In this study, a 2-sample MR method was adopted to evaluate the bidirectional causal relationship between 731 immunophenotypes and prostatitis, and the disease-related immunophenotypes were thus screened out. Then, the following 2 questions were explored thoroughly: Did the immune cells indirectly correlate with prostatitis by changing the levels of metabolites? Which metabolites were regulated by the immune cells to correlate with prostatitis? We employed 2-step MR and the pooled association statistics of immunophenotypes, metabolites, and prostatitis to estimate the mediation effects of 1400 metabolites and tested the hypothesis (Fig. 1).

### 2.2. Data sources

In the present study, the data from multiple cohorts were selected to investigate the relationship between the immunophenotypes and prostatitis. All GWAS data were from Europeans to avoid the bias from different races.

The data of the 731 immunophenotypes (ID: ebi-a-GCST90001391~ebi-a-GCST90002121) in this study were all from GWAS Catalog (https://www.ebi.ac.uk/gwas/) and had been acquired from 3757 Europeans (Sardinia, Italy), and there was no overlapping of cohorts.^[20]^ These phenotypes were classified into 7 groups: B cells, CDC, mature T cells, monocytes, bone marrow cells, TBNK (T, B, and natural killer cells), and Treg. The parameters included absolute count (n = 118), relative count (n = 192), median fluorescent intensity (MFI) reflecting the surface antigen level (n = 389), and morphological parameters (CDC and TBNK subsets; n = 32).

The pooled GWAS statistic data of 1400 plasma metabolites (ID: GCST90199621~GCST90201020) were derived from GWAS Catalog (https://www.ebi.ac.uk/gwas/), involving 1091 metabolites and 309 ratios from the samples collected from 8299 Europeans in the Canadian Longitudinal Study on Aging (CLSA).^[21]^

The data about prostatitis were acquired from the IEU Open GWAS website(https://gwas.mrcieu.ac.uk/), and the samples were collected from Europeans including 1859 patients with prostatitis, 72,799 healthy individuals without prostatitis, and 16,377,460 single nucleotide polymorphisms (SNPs).^[22]^ All the above-mentioned data were collected from publicly available literature and online websites and downloaded for free without any ethical restrictions.

### 2.3. Selection of instrumental variables (IVs)

To ensure the accuracy and reliability of the results, we strictly screened the SNPs. The IVs were selected based on the following criteria: with a strong correlation with the exposure; without correlations with confounding factors; affecting the outcome through the exposure only.^[23]^ Because the eligible IVs (genome-wide significance threshold, *P* < 5 × 10^−8^) were limited, the genome-wide significance was determined based on the threshold of *P* < 1 × 10^−5^ to ensure more comprehensive outcomes.^[24]^ Then, the aggregation method (*r*^2^ *=* 0.001, kb = 10,000) was used to eliminate linkage disequilibrium (LD). The palindromic SNPs were removed to avoid bias. Finally, variance (*R*^2^) and *F* statistics were adopted to estimate the intensity of the selected IVs.^[25]^ *R*^2^ = [2 × β^2^ × EAF × (1 − EAF)]/[2 × β^2^ × EAF × (1 − EAF) + 2 × SE^2^ × N × EAF × (1 − EAF)], *F* = [*R*^2^ × (N − 2)]/(1 − *R^2^*), β represents the effect size of the SNP on the phenotype, N represents the sample size, EAF represents the effect allele frequency, and SE represents the standard error of the β value. There was substantial evidence of weak instrument bias removal that the *F* value was 10.^[26]^

### 2.4. Statistical analysis

MR was performed in RStudio 4.3.3 with the packages including “TwoSampleMR,” “vroom,” “dplyr,” “tidyr,” “VariantAnnotation,” “gwasglue,” “MRPRESSO” and “forestploter.” Five MR methods were used to evaluate the overall causal relationship between 731 immunophenotypes and prostatitis, as well as the mediation effect of the metabolites. The 5 methods were inverse variance weighting (IVW), MR-Egger, weighted median, weighted mode, and simple mode.

MR-Egger was mainly used to evaluate horizontal pleiotropy, and the model intercept close to 0 indicated no statistical difference (*P* > .05), that is, absence of horizontal pleiotropy.^[27]^ In the case that horizontal pleiotropy was excluded, IVW became the most precise and the most common method for estimating the causal relationship in 2-sample MR, and *P* < .05 suggested a significant correlation between the exposure and the outcome.^[28]^ Furthermore, a heterogeneity test was carried out for the data. In the case of the data being heterogeneous (*P* < .05), the IVW random effects model was chosen, or the fixed effect model was chosen to ensure reliable results.^[29]^ In addition, weighted median, weighted mode, and simple mode were mainly used to supplement the analysis method and acquire the evaluation results of orientation consistency (beta and OR value), thereby improving the reliability of the results.^[30–32]^ The MR-PRESSO method and leave-one-out method for sensitivity analysis were adopted during the MR analysis. MR-PRESSO was used for the outlier test, which could identify the potential outliers with horizontal pleiotropy. If an outlier (*P* < .05) was identified, it should be eliminated before reanalysis.^[33]^ The leave-one-out method for sensitivity analysis was employed to estimate the meta effect of the remaining SNPs after SNPs were eliminated one by one, followed by observation of the change in the overall results. The results were considered robust in the case of no significant change in the overall error bar after elimination of each SNP.^[34]^

### 2.5. Mediation analysis

The bidirectional 2-sample analysis was performed to assess the causal relationship between the immunophenotypes (exposure) and the prostatitis (outcome) and obtain the immunophenotypes with positive results. Subsequently, reverse MR analysis was performed with the positive immunophenotypes as the outcomes and prostatitis as the exposure, and the biases with a reverse causal relationship were eliminated (*P* < .05) before the yielding of the final positive results. The causal relationship between 1400 metabolites and prostatitis was evaluated to obtain the metabolites with positive results. The mediation analysis was conducted with the first step to obtain the effect of the exposure (a positive immunophenotype) on the mediator (a positive metabolite; beta1), the second step to reveal the effect of the mediators screened out in the first step (as the exposure) on the outcome (prostatitis; beta2), and the third step to adopt the cause-effect between the immunophenotypes (exposure) screened out in the first step and the prostatitis (outcome) as the total effect (betaAll). According to the rule of products of coefficients,^[35,36]^ the product between the 2 (beta1* beta2) served as the mediation effect, that is, as the indirect effect of the exposure on the outcome. The calculation formula of mediation (%) was beta1* beta2/betaAll.

### 2.6. Bayesian-weighted Mendelian randomization

To avoid bias of polygenic structure and pleiotropy induced by complex traits of the immunophenotypes, metabolites, and prostatitis, we employed Bayesian-weighted Mendelian randomization (BWMR) to verify the IVW results. BWMR not only considered the polygene-derived weak effect and the weakly horizontal pleiotropy but also adaptively detected abnormal values due to the significantly horizontal pleiotropy. In addition, BWMR was efficient and stable in the statistical analysis, thus ensuring the accuracy of the final results.^[37,38]^

## 3. Results

### 3.1. Screening results of IVs

Based on the IV screening conditions set in the present study, 18,621 immunophenotype-related SNPs (*F* values within the range of 19.537–3159.289, Table S1, Supplemental Digital Content, http://links.lww.com/MD/N706) and 34,843 metabolite-related SNPs (*F* values within the range of 19.503–5308.355, Table S2, Supplemental Digital Content, http://links.lww.com/MD/N706) were screened out. The reverse MR analysis yielded 20 prostatitis-related SNPs (*F* values within the range of 19.590–30.004, Table S3, Supplemental Digital Content, http://links.lww.com/MD/N706).

### 3.2. Relationship between immune cells and prostatitis

**Figure 2.**
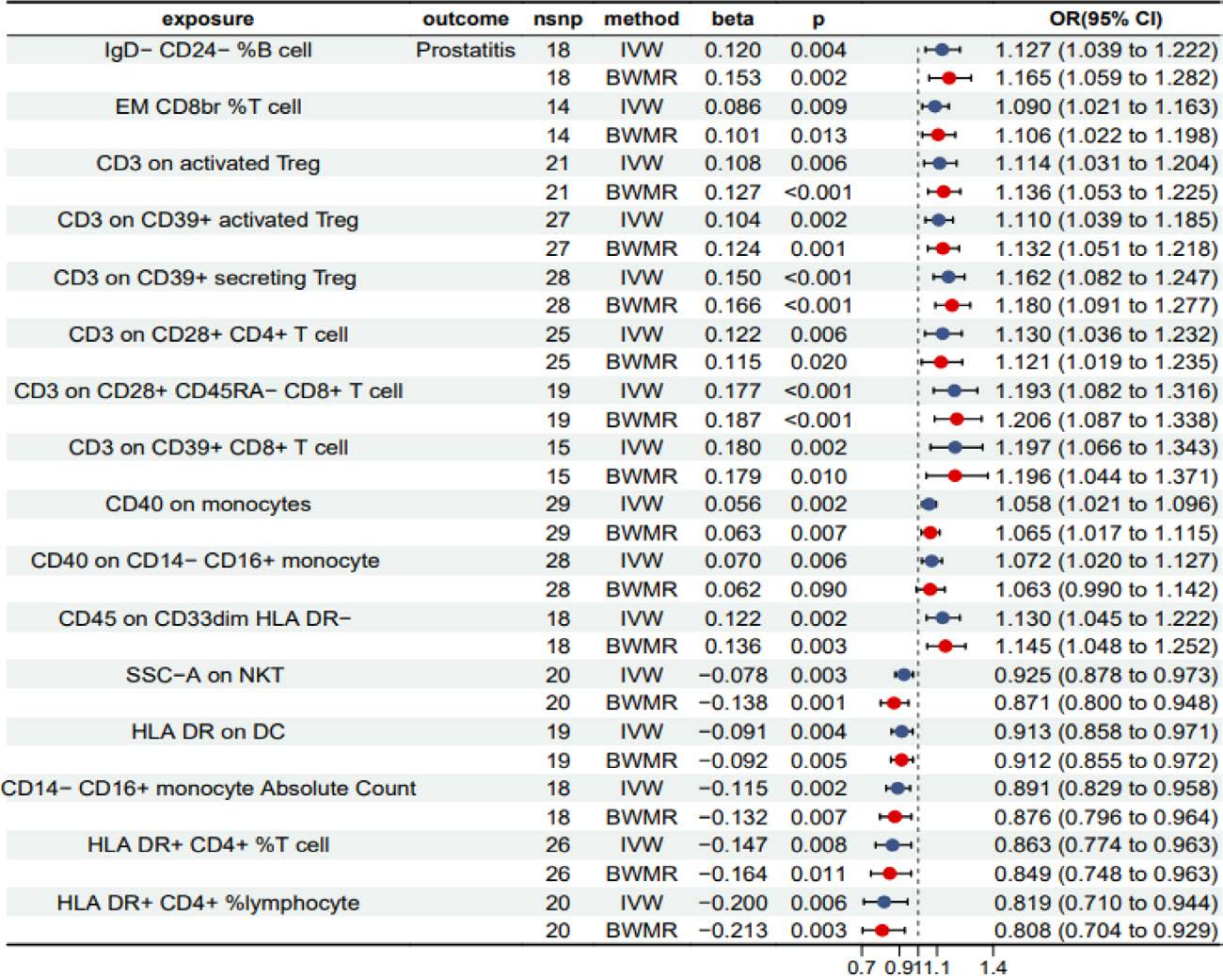
Forest plot to estimate the causal association between immunophenotypes and prostatitis by IVW and BWMR methods. beta = the effect size of the effect allele, BWMR = Bayesian weighted Mendelian randomization, CI = confidence interval, IVW = inverse variance weighted, nsnp = number of single nucleotide polymorphisms, OR = odds ratio.

To acquire more reliable and accurate results, we screened the IVs of immunophenotypes based on the condition of *P* < .01 and verified the results of IVW by the BMWR method after bidirectional MR analysis. A total of 16 prostatitis-related positive immunophenotypes were identified (Table S4, Supplemental Digital Content, http://links.lww.com/MD/N706, Fig. 2), including 11 immunophenotypes showing positive correlations: IgD^−^ CD24^−^ %B cell (IVW beta = 0.120, *P* = .004, 95% CI: 1.039–1.222), EM CD8br %T cell (IVW beta = 0.086, *P* = .009, 95% CI: 1.021–1.163), CD3 on activated Treg (IVW beta = 0.108, *P* = .006, 95% CI: 1.031–1.204), CD3 on CD39^+^ activated Treg (IVW beta = 0.104, *P* = .002, 95% CI: 1.039–1.185), CD3 on CD39^+^ secreting Treg (IVW beta = 0.150, *P* < .001, 95% CI: 1.082–1.247), CD3 on CD28^+^ CD4^+^ T cell (IVW beta = 0.122, *P* = .006, 95% CI: 1.036–1.232), CD3 on CD28^+^ CD45RA^−^ CD8^+^ T cell (IVW beta = 0.177, *P* < .001, 95% CI: 1.082–1.316), CD3 on CD39^+^ CD8^+^ T cell (IVW beta = 0.180, *P* = .002, 95% CI: 1.066–1.343), CD40 on monocytes (IVW beta = 0.056, *P = *.002, 95% CI: 1.021–1.096), CD40 on CD14^−^ CD16^+^ monocyte (IVW beta = 0.070, *P* = .006, 95% CI: 1.020–1.127)and CD45 on CD33dim HLA DR − (IVW beta = 0.122, *P* = .002, 95% CI: 1.045–1.222). In addition, 5 immunophenotypes showing negative correlations with prostatitis, including SSC − A on NKT (IVW beta = −0.078, *P* = .003, 95% CI: 0.878–0.973), HLA DR on DC (IVW beta = −0.091, *P* = .004, 95% CI: 0.858–0.971), CD14^−^ CD16^+^ monocyte absolute count (IVW beta = −0.115, *P* = .002, 95% CI: 0.829–0.958), HLA DR^+^ CD4^+^ %T cell (IVW beta = −0.147, *P* = .008, 95% CI: 0.774–0.963) and HLA DR^+^ CD4^+^ % lymphocyte(IVW beta = −0.200, *P* = .006, 95% CI: 0.710–0.944).

### 3.3. Relationship between metabolites and prostatitis

**Figure 3.**
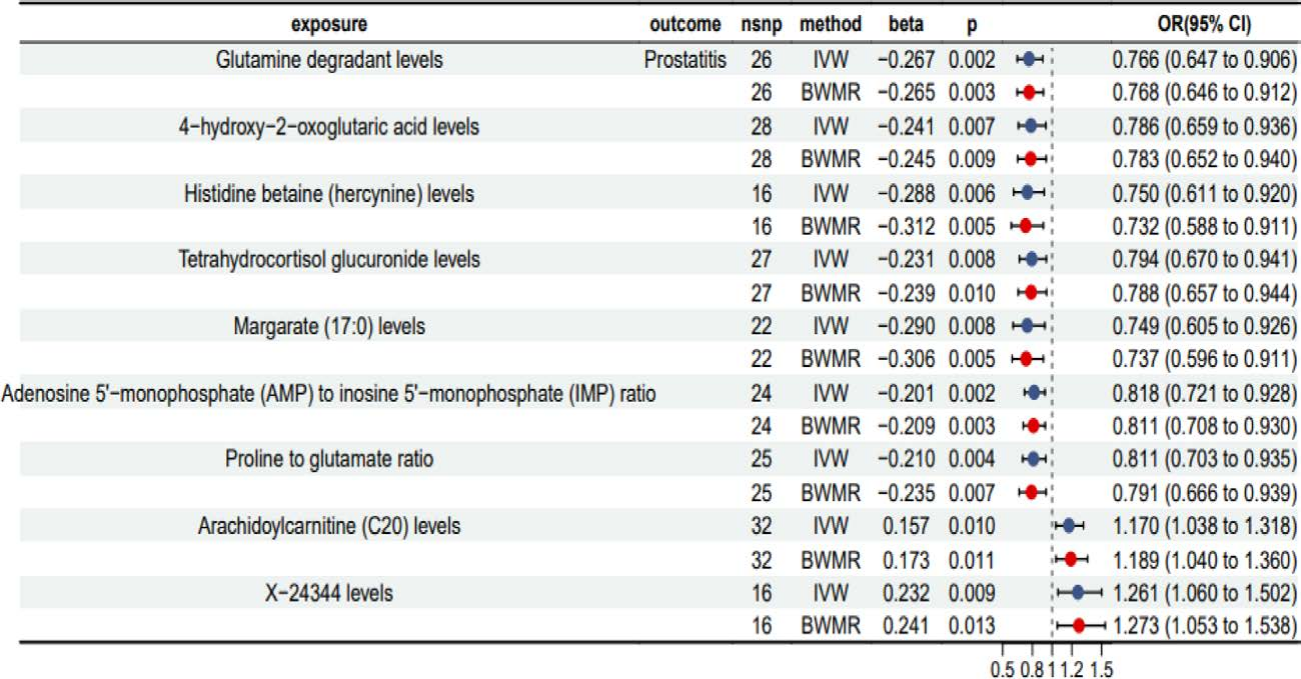
Forest plot to estimate the causal association between metabolites and prostatitis by IVW and BWMR methods. beta = the effect size of the effect allele, BWMR = Bayesian weighted Mendelian randomization, CI = confidence interval, IVW = inverse variance weighted, nsnp = number of single nucleotide polymorphisms, OR = odds ratio.

To avoid a false positives, we screened the IVs of plasma metabolites according to the condition of *P* < .01. Nine prostatitis-related metabolites were identified by MR and BWMR analyses (Table S5, Supplemental Digital Content, http://links.lww.com/MD/N706, Fig. 3), including 2 metabolites were associated with a positive risk of prostatitis: Arachidoylcarnitine (C20) levels (IVW beta = 0.157, *P* = .010, 95% CI: 1.038–1.318) and X − 24344 levels (IVW beta = 0.232, *P* = .009, 95% CI: 1.060–1.502). Whereas, 7 metabolites demonstrating negative correlations with prostatitis: glutamine degradant levels (IVW beta = −0.267, *P* = .002, 95% CI: 0.647–0.906), 4 − hydroxy − 2 − oxoglutaric acid levels (IVW beta = −0.241, *P* = .007, 95% CI: 0.659–0.936), histidine betaine (hercynine) levels (IVW beta = −0.288, *P* = .006, 95% CI: 0.611–0.920), tetrahydrocortisol glucuronide levels (IVW beta = −0.231, *P* = .008, 95% CI: 0.670–0.941), Margarate (17:0) levels (IVW beta = −0.290, *P* = .008, 95% CI: 0.605–0.926), adenosine 5′−monophosphate (AMP) to inosine 5′−monophosphate (IMP) ratio (IVW beta =−0.201, *P* = .002, 95% CI: 0.721–0.928) and proline to glutamate ratio (IVW beta =−0.235, *P* = .007, 95% CI: 0.666–0.939).

### 3.4. Reverse MR analysis

**Figure 4.**
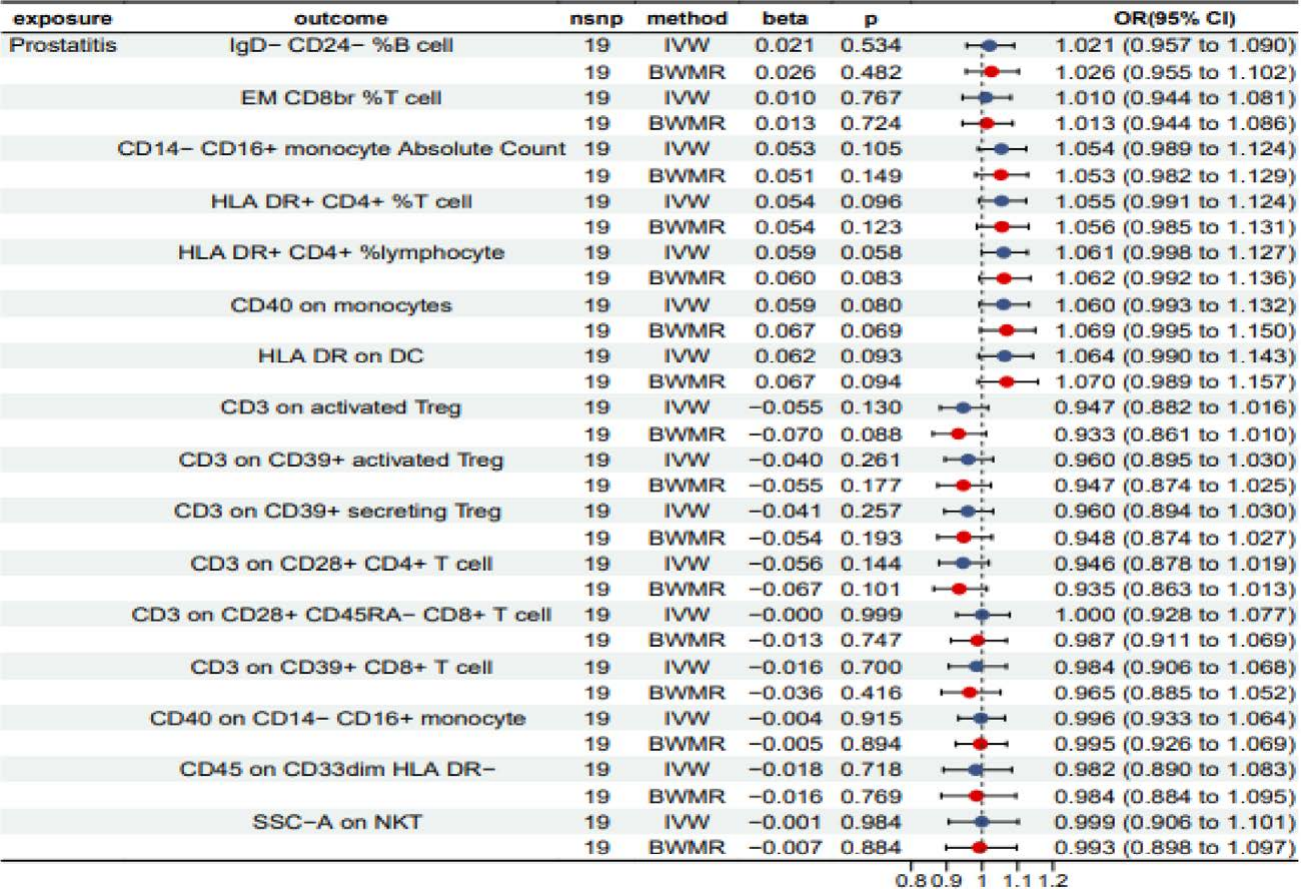
The results of the reverse MR and BWMR methods estimated for the association between prostatitis and immunophenotypes. beta = the effect size of the effect allele, BWMR = Bayesian weighted Mendelian randomization, CI = confidence interval, IVW = inverse variance weighted, nsnp = number of single nucleotide polymorphisms, OR = odds ratio.

The reverse MR analysis on the causal relationship between immunophenotypes and prostatitis showed no bias for reverse causal relationship of the 16 positive immunophenotypes (Table S6, Supplemental Digital Content, http://links.lww.com/MD/N706). To ensure the reliability of the results, we adopted the BMWR method to verify the IVW results, which were demonstrated to be consistent (Fig. 4, orientation consistency of beta, *P* > .05). Additionally, we also analyzed the reverse causality between these 9 metabolites and prostatitis, and the results were all negative (IVW *P* > .05, Table S7, Supplemental Digital Content, http://links.lww.com/MD/N706).

### 3.5. Metabolite-mediated causal relationship between immune cells and prostatitis

**Figure 5.**
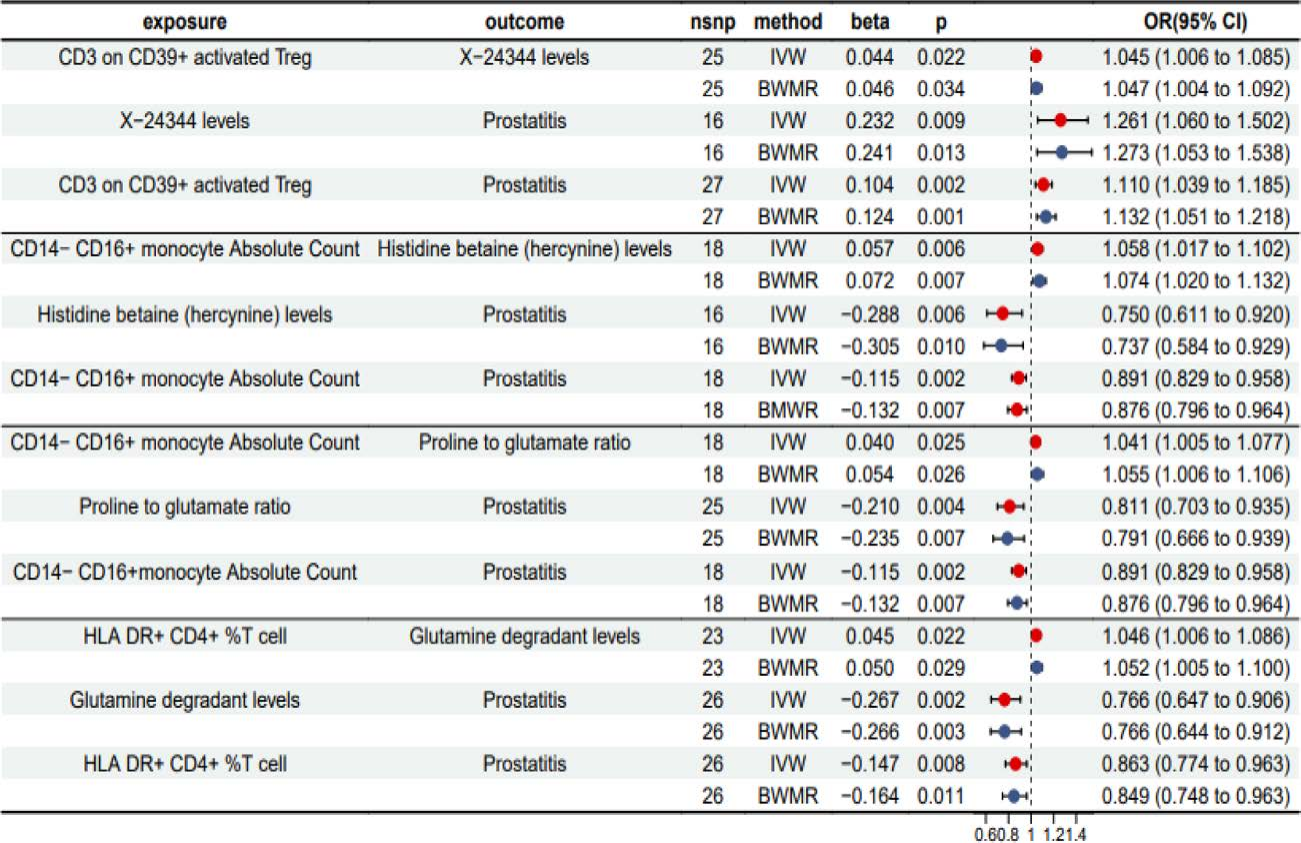
Forest plot to show the mediation analysis of metabolites modulation of immunophenotypes affecting prostatitis. beta = the effect size of the effect allele, BWMR = Bayesian weighted Mendelian randomization, CI = confidence interval, IVW = inverse variance weighted, nsnp = number of single nucleotide polymorphisms, OR = odds ratio.

We performed MR analysis on the causal relationship between the exposure and the medium (beta1) by using the metabolite as the mediator and adopted BWMR to verify the results (Table S8, Supplemental Digital Content, http://links.lww.com/MD/N706, Fig. 5.). Similarly, we evaluated the effect of the medium on the outcome (beta2; Table S5, Supplemental Digital Content, http://links.lww.com/MD/N706, Fig. 5.) and the total effect (betaAll) of the exposure on the outcome (Table S4, Supplemental Digital Content, http://links.lww.com/MD/N706, Fig. 5.). Four metabolites as the mediators were involved in the causal relationship between 3 immunophenotypes and prostatitis. CD3 on CD39^+^ activated Treg influenced the risk of prostatitis via the X-24344 levels; the absolute count of CD14^–^ CD16^+^ monocytes affected the risk of prostatitis via the histidine betaine (hercynine) levels and the proline-to-glutamate ratio; and HLA-DR^+^ CD4^+^ %T cells affected the prostatitis risk via the glutamine degradant levels.

According to the results of the mediation effect (Table 1), the following conclusions were drawn: The indirect effect (mediation effect) value of X-24344 levels in mediating the pathway of CD3 on CD39^+^ activated Treg to prostatitis was 0.01, with a ratio of 9.82%; histidine betaine (hercynine) levels and the proline-to-glutamate ratio were involved in the relationship between the absolute count of CD14^−^ CD16^+^ monocytes and prostatitis, with the indirect effect value being − 0.016 (14.20%) and − 0.008 (7.24%), respectively; The indirect effect value of glutamine degradant levels in the causal relationship between HLA DR^+^ CD4^+^ %T cell and prostatitis was −0.012, accounting for 8.07% of the total. All these results showed statistical differences (*P* < .01).

**Table 1:** Mediation effect of metabolites in the association between immunophenotypes and prostatitis.

Exposure	Mediator	Outcome	*P* value	Mediated effect	95% CI	Mediated proportion (%)
CD3 on CD39^+^ activated Treg	X-24344 levels	Prostatitis	.023	0.010	0.0014 to 0.0191	9.82
CD14^−^ CD16^+^ monocyte AC	Histidine betaine (hercynine) levels	Prostatitis	.007	−0.016	−0.0045 to −0.0282	14.20
CD14^−^ CD16^+^ monocyte AC	Proline to glutamate ratio	Prostatitis	.027	−0.008	−0.0009 to −0.0158	7.24
HLA DR^+^ CD4^+^ %T cell	Glutamine degradant levels	Prostatitis	.024	−0.012	-0.0016 to −0.0222	8.07

Mediated effect = beta1 × beta2, mediated proportion = beta1 × beta2 / betaAll.

### 3.6. Sensitivity analysis

In the sensitivity analysis of the causal relationship between immunophenotypes or metabolites and prostatitis, the intercept of MR-Egger regression had no deviation (0; *P* > .05), suggesting an absence of horizontal pleiotropy (Tables S9–S11, Supplemental Digital Content, http://links.lww.com/MD/N706). The heterogeneity test revealed no heterogeneity in the 16 positive immunophenotypes, 9 metabolites, or causal analysis process (Cochrane *Q* test for IVW: *P* > .05, Tables S12–S14, Supplemental Digital Content, http://links.lww.com/MD/N706), and thus the fixed effect model was selected. The MR-PRESSO test showed no outlier (*P* > .05) in the data (Tables S15–S17, Supplemental Digital Content, http://links.lww.com/MD/N706). The leave-one-out analysis (Figs. S1–S3, Supplemental Digital Content, http://links.lww.com/MD/N707, http://links.lww.com/MD/N708, http://links.lww.com/MD/N709.) and funnel plots (Figs. S4–S6, Supplemental Digital Content, http://links.lww.com/MD/N710, http://links.lww.com/MD/N711, http://links.lww.com/MD/N712.) indicated no specific SNP significantly influencing the causal relationship, which verified the robustness of the MR results.

## 4. Discussion

In the present study, the MR method was used to analyze the causal relationship between immune cells and prostatitis from the perspective of genetics. A total of 16 immunophenotypes were found to be involved in the occurrence of prostatitis, including 11 immunophenotypes increasing the risk of prostatitis. Wherein, 8 immune cell subsets were mature T cells and expressed CD3. This result was consistent with that from the observational study conducted by Zhang et al,^[6]^ that is, the patients with prostatitis presented significantly higher level of T cells than that those without prostatitis and a mass of CD3^+^ T cells. In addition, there were 2 subsets of monocytes expressing CD40, 1 subset of B cells, and 1 subset of myeloid cells, which were similar to the results from 2 observational studies. One study was conducted for 115 patients with chronic prostatitis by prostate biopsy, and the results showed significant increases in monocytes/macrophages, T cells, and B cells in the prostatitis region, suggesting the involvement of these cells in the occurrence of prostatitis.^[39]^ The other study was conducted with the prostatic fluid of such patients and showed that the counts of granulocytes, monocytes/macrophages, and T- and B-lymphocytes rose.^[40]^ Unfortunately, the 2 studies did not perform typing of the immune cells, thus failing to determine the specific immunophenotypes.

Interestingly, this study predicted that 5 immunophenotypes were negatively correlated with the risk of prostatitis, with the specific immunophenotypes being SSC-A on NKT, HLA-DR on DC, absolute count of CD14^–^ CD16^+^ monocytes, HLA-DR^+^ CD4^+^ %T cells, and HLA-DR^+^ CD4^+^ % lymphocytes, respectively, which might be involved in the negative regulation of prostatitis. Some scholars demonstrated in animal experiments that the symptoms of local inflammatory reactions and pain might be relieved by inducing monocytes/macrophages to aggregate inside the prostate gland, promoting the macrophage polarization toward the M2 phenotype, and increasing the proportion of anti-inflammatory M2 macrophages.^[41–43]^ The other studies showed that modeled rats had low NKT cell levels in the prostate gland and exhibited hereditary susceptibility to prostatitis,^[44]^ while healthy rats without prostatitis showed abnormally high proportions of NKT and NK cells in the prostate gland, with specific phenotypes identified: CD161adull^+^ αβTCR^+^ NKT, CD161a^+^ CD4^+^ αβTCR^+^ NKT, and CD161adull^+^ αβTCR-NK.^[45]^ The relationship between dendritic cells (DCs) and prostatitis pathogenesis has not been explored.^[46]^ Up to now, it has only been reported in 1 study that the production of tolerogenic DCs phenotypes could enhance the viability of CD4 + CD25 + suppressor T cells in the prostate tissue and maintain the tolerance of the prostate to autoantigens.^[47]^

From the 1400 metabolites used as the mediums, 9 prostatitis-related metabolites were screened out, including 4 metabolites (X-24344 levels, histidine betaine (hercynine) levels, proline-to-glutamate ratio, and glutamine degradant levels) involved in the total causal relationship between immune cells and prostatitis. Studies have identified multiple metabolic disorders in the rat model of nonbacterial prostatitis, including disruptions in histidine, nicotinate, nicotinamide, phenylalanine, tyrosine, arginine and proline metabolism. Some of these metabolic imbalances may be reversed by berberine, which significantly improves the pathological process and alleviates the symptoms of prostatitis in rats. Meanwhile, as some metabolic processes recover, the in vivo immune dysfunction and the inflammatory response in multiple organs improve, suggesting a possible role of metabolites in regulating immune imbalance in patients with prostatitis.^[48]^ However, this study did not further explore the specific mechanisms of immunoregulation involving these metabolites. In another study constructing the rat model of prostatitis, researchers detected several abnormal metabolic programs, including the biosynthesis of phenylalanine, tyrosine and tryptophan, as well as the metabolism of phenylalanine, tyrosine, arginine, proline, glycine, serine, threonine, alanine, aspartic acid and glutamic acid. Interventions targeting these metabolic pathways significantly relieved inflammation and impeded disease progression in the rat model.^[49]^ In patients with prostatitis, 20 plasm metabolites of high level were detected, including phenylpropanoids, organic heterocyclic compounds, organic acids and their derivatives, lipids, lipoids, organic oxygen compounds, and polypeptides.^[50]^

To our knowledge, this study was the first time that these metabolites serving as mediation factors for analyzing the possible influences of immune cells on prostatitis risk via indirect pathways. The NF-κB signaling pathway was essential to promote and maintain the inflammatory reaction of the prostate,^[43]^ and glutamic acid as the precursor of glutamine can suppress the NF-κB signaling pathway and the inflammatory reaction of the prostate.^[51,52]^ Moreover, glutamine serves as a nutrient in the prostate, and its degradation produces citrate to provide fuel for the tricarboxylic acid (TCA) cycle, thereby maintaining normal physiological function of the prostate.^[53]^ When the prostate was in inflammation, the levels of glutamic acid and glutamine declined, which hindered the TCA cycle and jeopardized the homeostasis of the prostate, thereby aggravating the inflammation and pain and causing depression.^[51]^

The CD4^+^ T cells included 2 subsets of Th17 and Treg, which possess opposite functions and were in immunologic balance in the case of a normal prostate environment.^[54]^ Th17 cells were a key to inducing and aggravating pelvic pain and male genital tract inflammation, while Treg played a role in suppressing inflammation, controlling autoimmunity, and maintaining immunity homeostasis.^[55]^ The functional defect of Treg cells might increase the susceptibility to chronic prostatitis,^[56]^ and the patients with chronic prostatitis had a low proportion of Treg cells^6^. The activation of CD4^+^ T cells accelerated the intake and metabolism of glutamine, the degradation of which induced the differentiation of CD4^+^ T cells to Th17/Treg cells so as to maintain the immunologic function of the cell subsets. The imbalance of the cell subsets would increase the risk of immune diseases.^[57]^ Animal experiments have validated that the differentiation of CD4^+^ T cells is depended on glutamine metabolism. When glutamine is catabolized with the aid of a catalyzer, it not only provides the energy necessary to activate CD4^+^ T cells but also produces decomposed products (glutamate and α-ketoglutarate) that directly induce the production of pro-inflammatory Th17 cell subpopulations and maintain their immunologic function. Conversely, when this metabolic process is deprived, the proportion of Th17 cells is significantly downregulated,^[58]^ and the transcription factor Foxp3 is upregulated, promoting Treg cell proliferation.^[14,59]^ Furthermore, mutual transformation may occur between the 2 phenotypes of Th17 and Treg cells, and the Th17/Treg cell homeostasis can be regulated directly by the glutamine-glutamic acid-GSH pathway, glutamine-glutamic acid-α-KG-2-HG pathway and glutamine-mTOR signal transduction pathway.^[57]^ This regulatory mechanism is also supported in patients with rheumatoid arthritis and systemic lupus erythematosus. Specifically, these patients exhibit an increased proportion of CD4^+^ T and Th17 cells, a decreased proportion of FoxP3^+^ Treg cells, and highly activated CD4^+^ T cells. These metabolic disorders lead to the release of numerous inflammatory factors and promote the uptake of glutamine and other major neutral amino acids (leucine, phenylalanine, and isoleucine) as well as the metabolic activity of glutamine.^[60,61]^

The results in this study showed that the indirect effect of glutamine degradant levels in mediating the causal relationship between HLA^–^DR^+^CD4^+^ %T cells and prostatitis was −0.012, accounting for 8.07% of the total (*P* = .024 < 0.05). Therefore, we hypothesized that CD4^+^ T cells might change the risk of prostatitis, which might be mediated by the metabolic pathway of glutamine. As shown in Figure 5, in the regulatory chain of “HLA^–^DR^+^CD4^+^ %T cells–Glutamine degradant levels–Prostatitis,” HLA^–^DR^+^CD4^+^ %T cells positively influence the metabolic pathway of glutamine (beta = 0.045 > 0), while this pathway negatively regulates the risk of prostatitis (beta = −0.267 < 0). These finding are consistent with the prevailing theory regarding glutamine metabolism and its impact on prostatitis. As for the mediation of X-24344 levels in the causal relationship between CD3 on CD39^+^ activated Treg and prostatitis and that of histidine betaine (hercynine) levels and proline-to-glutamate ratio in the causal relationship between CD14^–^CD16^+^ monocytes and prostatitis, no similar theories were reported in the available articles.

The present study adopted MR analysis combined with the BWMR method to investigate the causal relationship between immune cells and prostatitis and successfully constructed some pathways of immune cells acting on prostatitis via metabolites (as mediators). Compared with the conventional observational studies, our study was carried out based on the pooled large-scale GWAS dataset to eliminate as much as possible the bias of causal relationships and the interference of confounding factors, which ensured that the results were reliable. However, this study had the following limitations. All the data were collected from Europeans, which limited the applicability of the conclusion to other populations. Confounding factors and horizontal pleiotropy cannot be eliminated. Fundamental experiments and clinical studies needed to be carried out for further verification.

## 5. Conclusion

In short, the possible pathways for the causal relationship between immune cells or plasma metabolites and prostatitis were uncovered from genetics. Deciphering the potential key mechanisms and mining the targets in the relationships of prostatitis with immune cells and plasma metabolites helps to recover the immune microenvironment homeostasis, which can be regarded as a novel therapeutic strategy for prostatitis.

## Author contributions

**Conceptualization:** Chao Ding.

**Data curation:** Chao Ding.

**Formal analysis:** Chao Ding.

**Funding acquisition:** Chao Ding, Shui Wan.

**Investigation:** Chao Ding.

**Methodology:** Chao Ding.

**Project administration:** Chao Ding.

**Resources:** Chao Ding.

**Software:** Chao Ding.

**Supervision:** Quanhua Gong, Shui Wan.

**Validation:** Shui Wan.

**Visualization:** Chao Ding.

**Writing – original draft:** Chao Ding.

**Writing – review & editing:** Chao Ding, Quanhua Gong, Shui Wan.

## Supplementary Material


