# Supplementary figures and images for: Mediation effect of plasma metabolites on the relationship between immune cells and the risk of prostatitis: A study by bidirectional 2-sample and Bayesian-weighted Mendelian randomization
Source: Medicine (Baltimore). 2024 Oct 11;103(41):e40024. doi: 10.1097/MD.0000000000040024 (PMC11479442; doi:10.1097/MD.0000000000040024)

## Slide 1
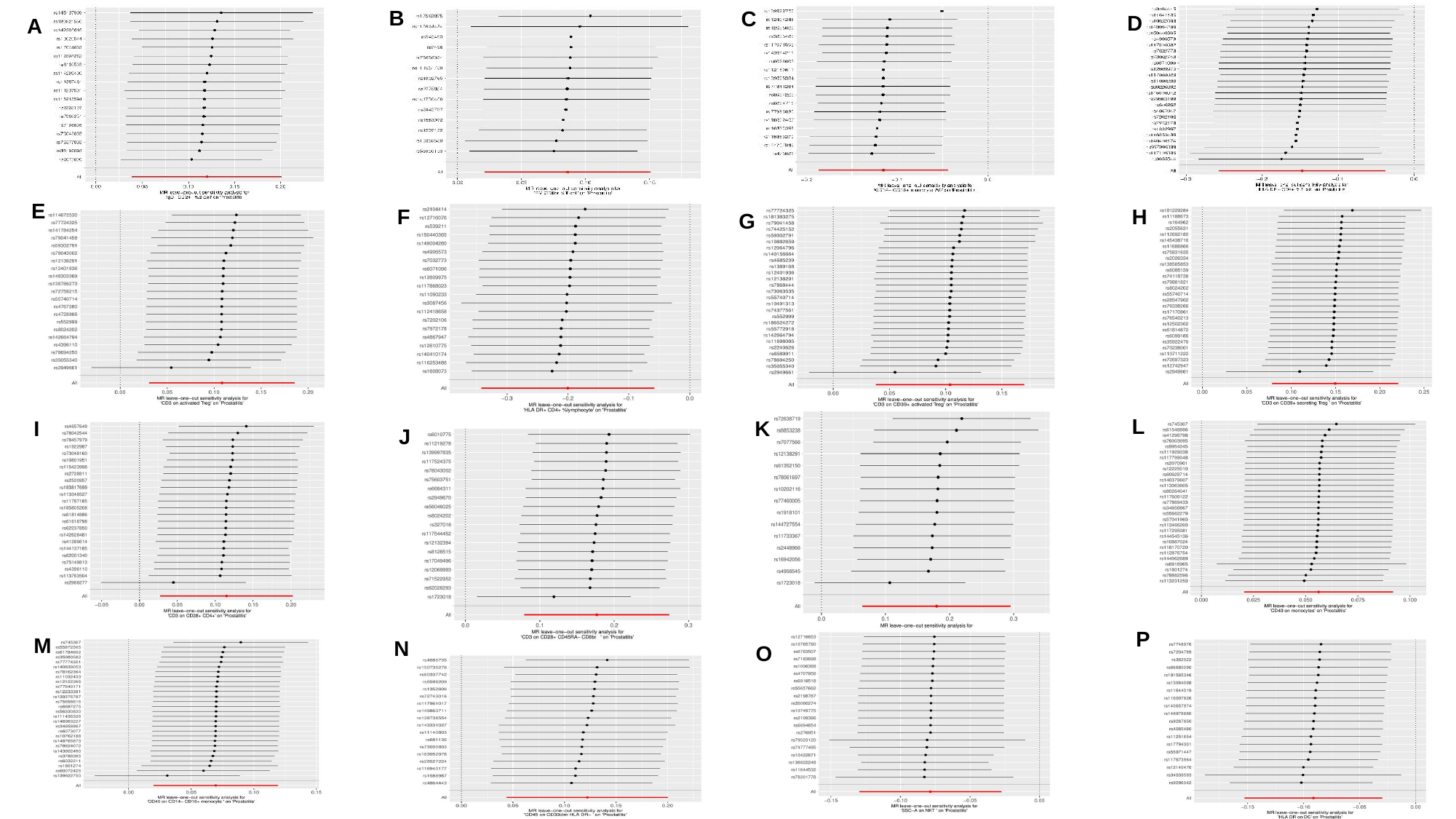

B
C
D
A
E
F
H
G
L
I
K
J
P
M
N
O

Supplement: Supplementary file 2 [file medi-103-e40024-s002.pptx]

## Slide 1
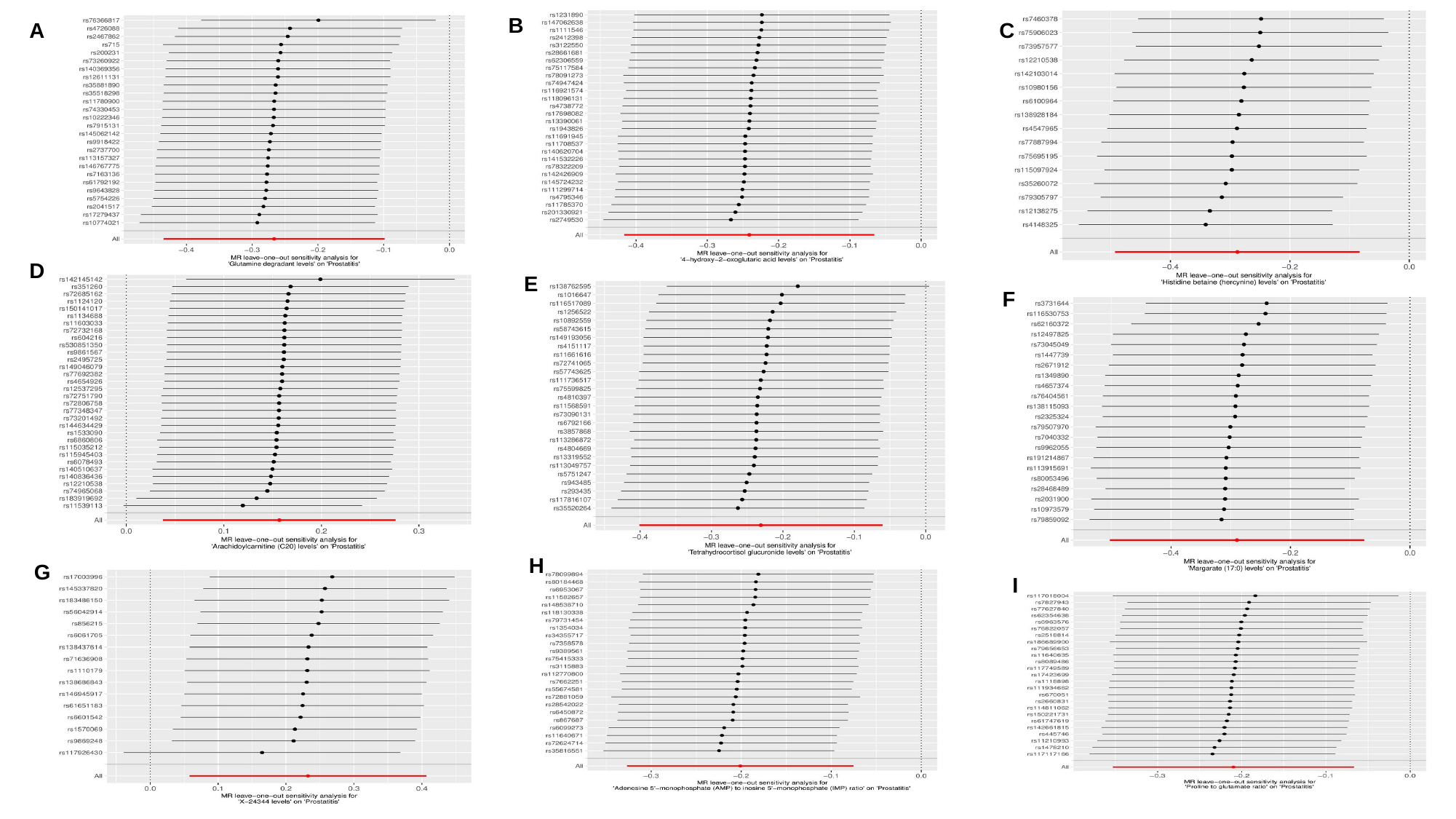

B
A
C
D
E
F
H
G
I

Supplement: Supplementary file 3 [file medi-103-e40024-s003.pptx]

## Slide 1
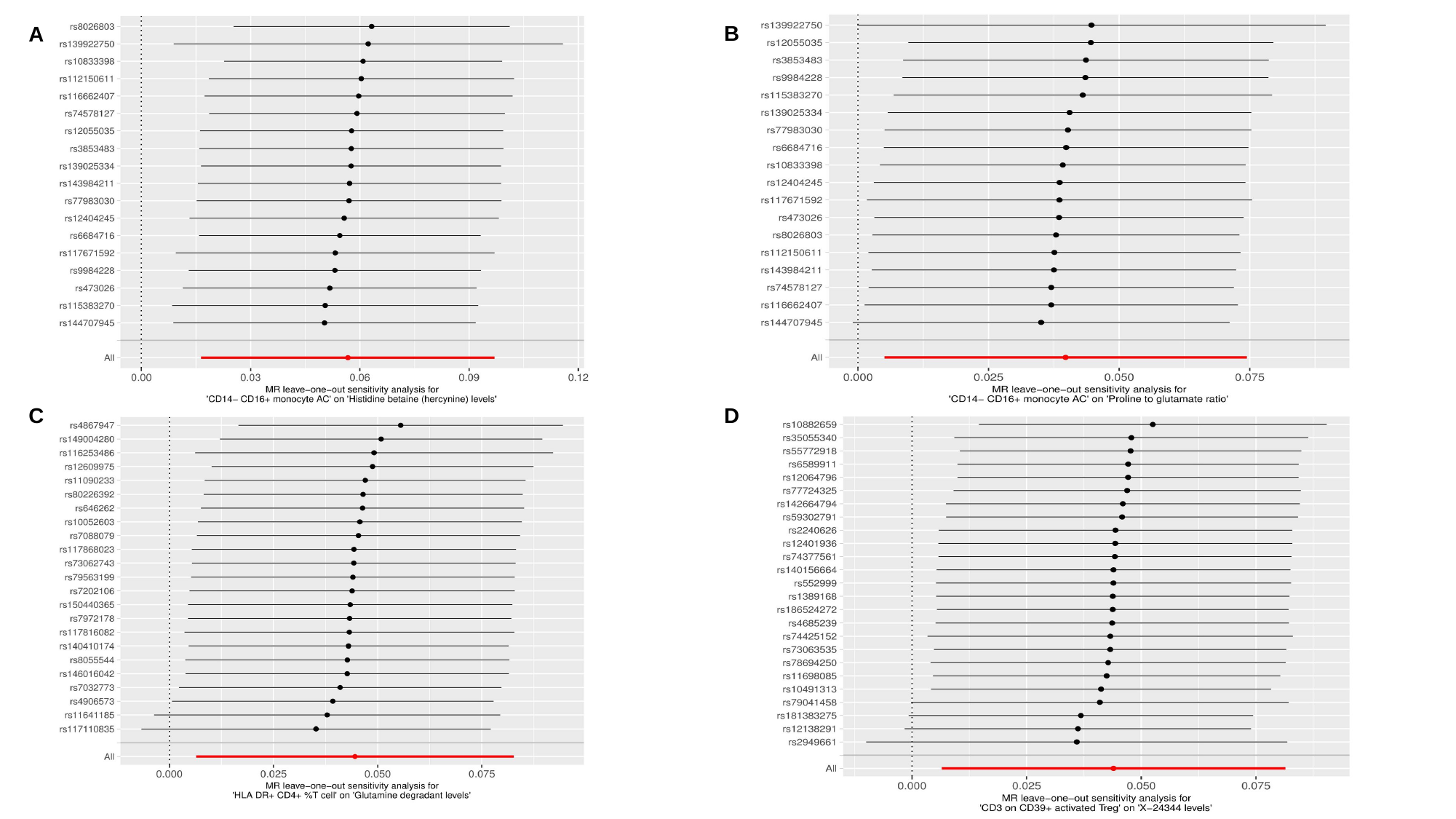

B
A
D
C

Supplement: Supplementary file 4 [file medi-103-e40024-s004.pptx]

## Slide 1
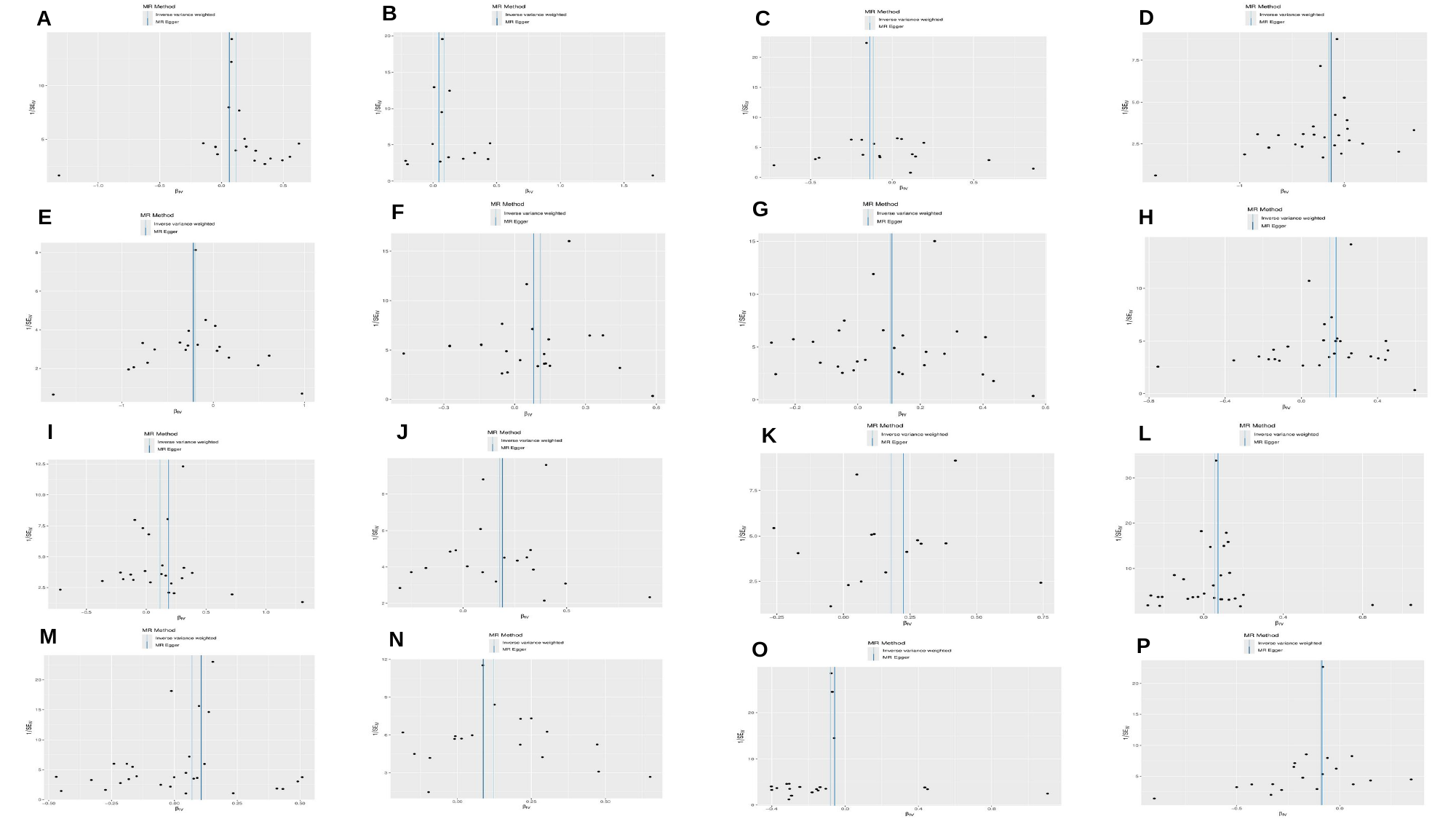

B
D
C
A
G
F
E
H
I
J
L
K
M
N
P
O

Supplement: Supplementary file 5 [file medi-103-e40024-s005.pptx]

## Slide 1
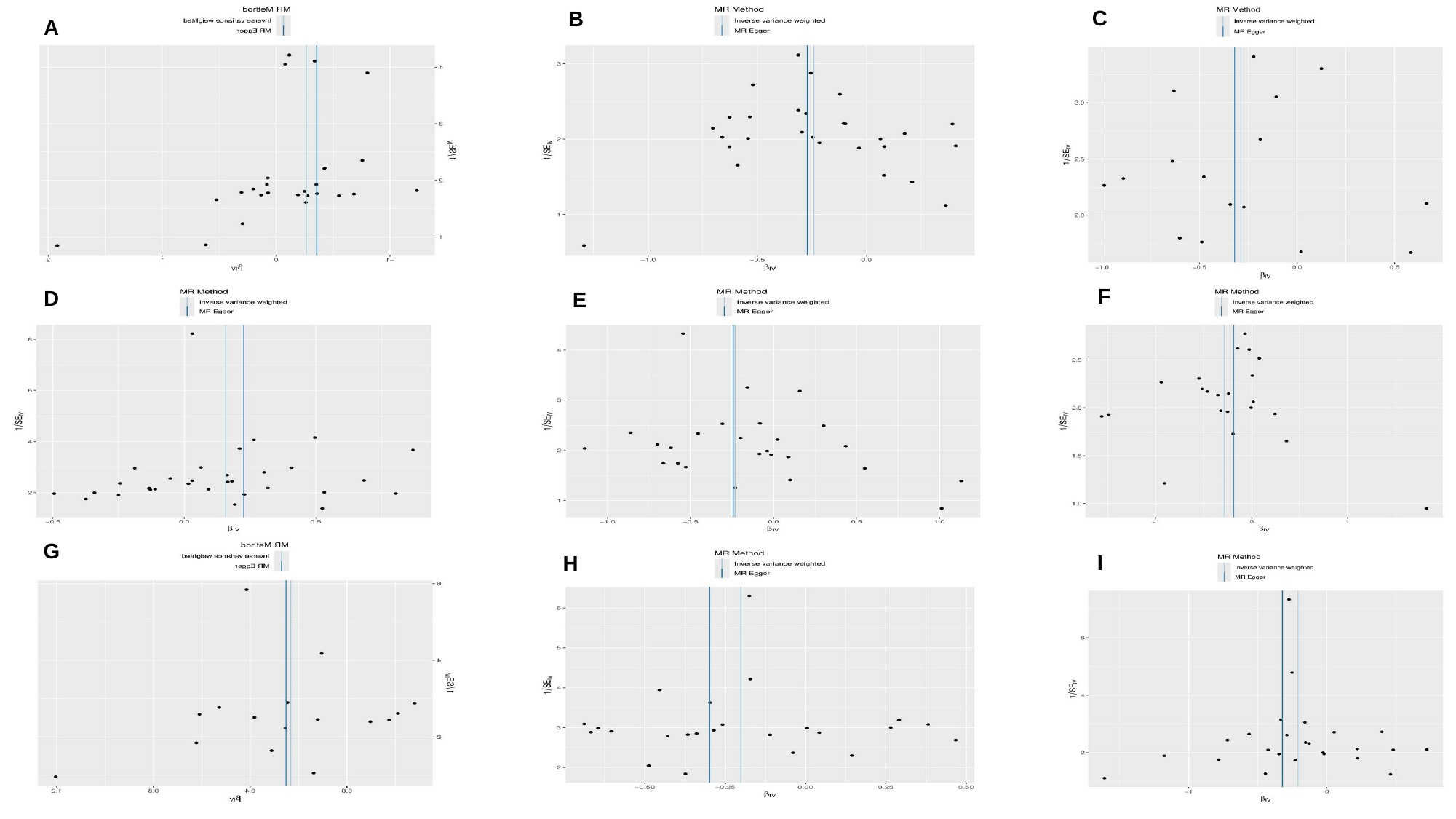

B
C
A
F
D
E
G
I
H

Supplement: Supplementary file 6 [file medi-103-e40024-s006.pptx]

## Slide 1
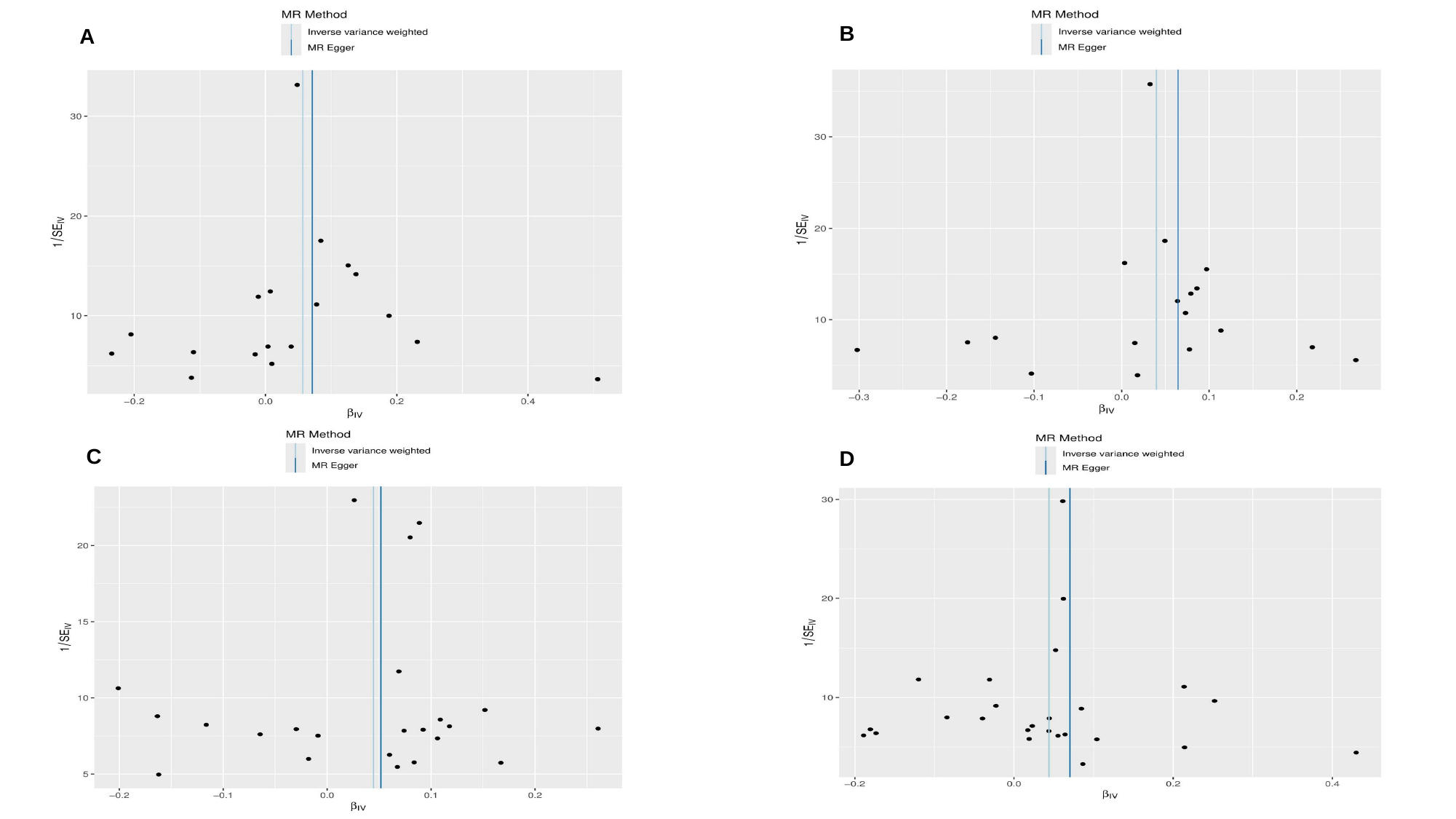

B
A
C
D

Supplement: Supplementary file 7 [file medi-103-e40024-s007.pptx]
